# Late cholestatic syndrome due to previous perforating trauma: Case report

**DOI:** 10.1016/j.ijscr.2019.07.073

**Published:** 2019-08-01

**Authors:** Frank Pinheiro Pessoa Coelho De Macedo, Carolina Augusta Dorgam Maués, Otávio Mendes Filho, Ketlen Gomes da Costa, Juan Eduardo Rios Rodriguez, Irma Csasznik, João José Corrêa Bergamasco, Rubem Alves da Silva Neto, Rubem Alves da Silva Júnior

**Affiliations:** a Digestive System Surgery Service at Getúlio Vargas Teaching Hospital (HUGV), Avenida Apurinã, 4 - Praça 14 de Janeiro, Manaus, Amazonas, 69020-170, Brazil; b General Surgery Service at Getúlio Vargas Teaching Hospital (HUGV), Avenida Apurinã, 4 - Praça 14 de Janeiro, Manaus, Amazonas, 69020-170, Brazil; c Medical School of Federal University of Amazonas (UFAM), Rua Afonso Pena, 1053 - Praça 14 de Janeiro, Manaus, Amazonas, 69020-160, Brazil; d Getúlio Vargas Teaching Hospital (HUGV), Avenida Apurinã, 4 - Praça 14 de Janeiro, Manaus, Amazonas, 69020-170, Brazil

**Keywords:** Cholestasis, Cholangitis, Foreign bodies, Foreign-body migration, Gunshot wound, Obstructive jaundice

## Abstract

**Introduction:**

The presence of a foreign body obstructing the bile duct may appear through a typical picture of cholangitis, usually caused by the presence of gallstones.

**Presentation of case:**

Male patient, 31 years old, involved an in accident with gunshot in 2006, injured in the abdominal region. Evolved to a picture of intense pain in the right hypochondrium, mainly after fat meals, without irradiation 12 years after being injured. Presented jaundice episode associated to fecal acholia, choluria, pruritus and sporadic fever. It was diagnosed cholangitis due to the foreign body like a bullet, located in the hepatic duct after tomography performed for investigation. The conduction of the case is described next.

**Discussion:**

Cholestatic syndrome caused by gun bullet in a prior event to the symptoms is a rare event in the literature. Total, there are 8 case reports of bullets found in bile ducts with distinct evolution time among themselves. The first exam will not necessarily identify the bullet, but it simplifies the visualization of the bile ducts dilation, characterizing obstruction.

**Conclusion:**

The presence of a foreign body in the bile ducts is well reported in literature, and it is directly dependent on previous history. We should always avoid using cholangioresonance in dubious cases. To keep the ERCP as initial treatment is recommended due to the advances on the procedure quality, but in more severe cases or lack of resources, the surgical exploration is still indicated.

## Highlights

• Bullets are rare causes of obstructive jaundice.
• Clinical examination is the most important step in diagnostic.
• Cholangioresonance is not safe in supected bullet obstruction.

## 1 Introduction

The presence of a foreign body obstructing the bile duct may appear through a typical picture of cholangitis, usually caused by the presence of gallstones. One of the causes of gallstone formation is the presence of a foreign body, such as surgical material (threads, endoclips, gauze), structures or food that came back from Vater’s ampulla or that migrated directly from the common bile duct or via transmural to the bile ducts [1].

Frequently, the detection of abnormality only occurs when the patient presents signs and symptoms of obstruction, not always evolving with icterus. There are cases in which the picture has mimicked a clinical picture of appendicitis [2]. The incubation period to present symptoms depends on the initial location of the foreign body, its entry mechanism, dimensions and composition, organic or inorganic [3,4].

We are going to report here an obstruction case of common hepatic duct, evolving to cholestatic syndrome due to a firearm bullet in the patient’s body that occurred 12 years before. This work has been reported in line with the SCARE criteria [5].

## 2 Report

Male patient, 31 years old, involved an in accident with gun in 2006, injured in the abdominal region, declares being in hospital for 5 days in the municipality of Presidente Dutra, countryside of Maranhão, and submitted to exploratory laparotomy surgery without bullet removal. He alleges a good evolution, immediate and late postoperative, without complications and asymptomatic.

In April of 2018, 12 years after being injured, evolved to a picture of intense pain in the right hypochondrium, mainly after hyperlipidic meals, without irradiation, presenting relief after taking scopolamine and dipyrone. Started medical follow up and was submitted to treatment for H. pylori detected in endoscopy, without presenting total improvement from the initial symptoms. Presented jaundice episode in August of 2018, associated to fecal acholia, choluria, pruritus and sporadic fever of approximately 38 °C, without specific time of the day.

**Fig. 1:**
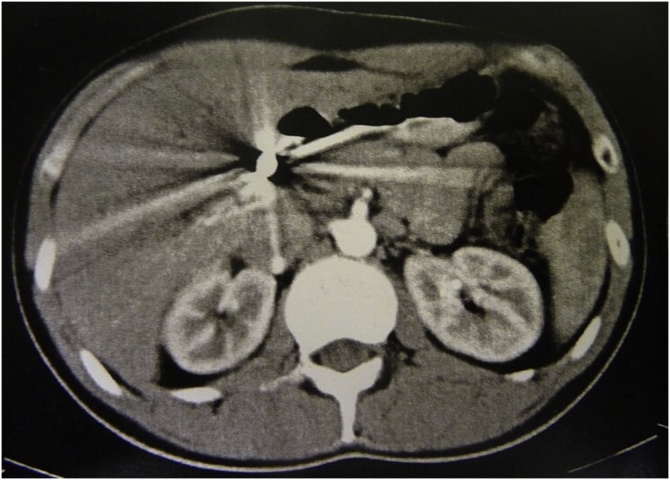
Computed tomography (CT) with visualization of metallic fragment near at biliary tract and hepatic hilum.

**Fig. 2:**
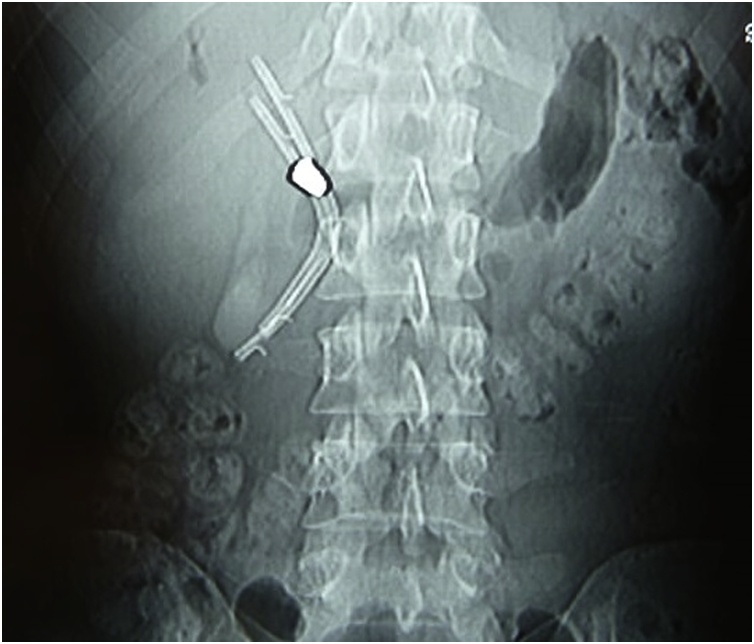
Control radiography after ERCP with the biliary duct stent.

It was diagnosed cholangitis due to the foreign body like a bullet, located in the hepatic duct after tomography performed for investigation (Fig. 1). The patient was referred to the elective surgery service for confirmation and performance of endoscopic retrograde cholangiopancreatography (ERCP) in September of 2018. During the procedure, two plastic prosthesis were installed in bile ducts for dilation, because the bullet had not been reached due to its disproportion in comparison with the bile duct. Also, radiography was realized just after the procedure (Fig. 2). Evolved post CPRE with acute pancreatitis properly treated. He describes that since the period when the cholangitis picture started up to the moment (7 months later) presented weight loss of 20 kg.

**Fig. 3:**
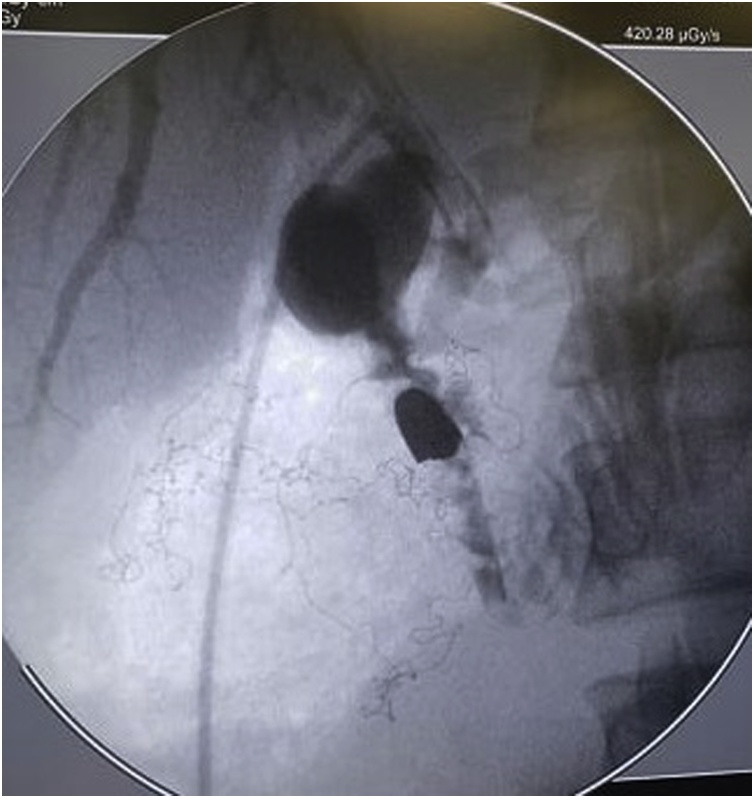
Intraoperative Cholangiography examination evidencing ballistic projectile inside of biliary tract.

**Fig. 4:**
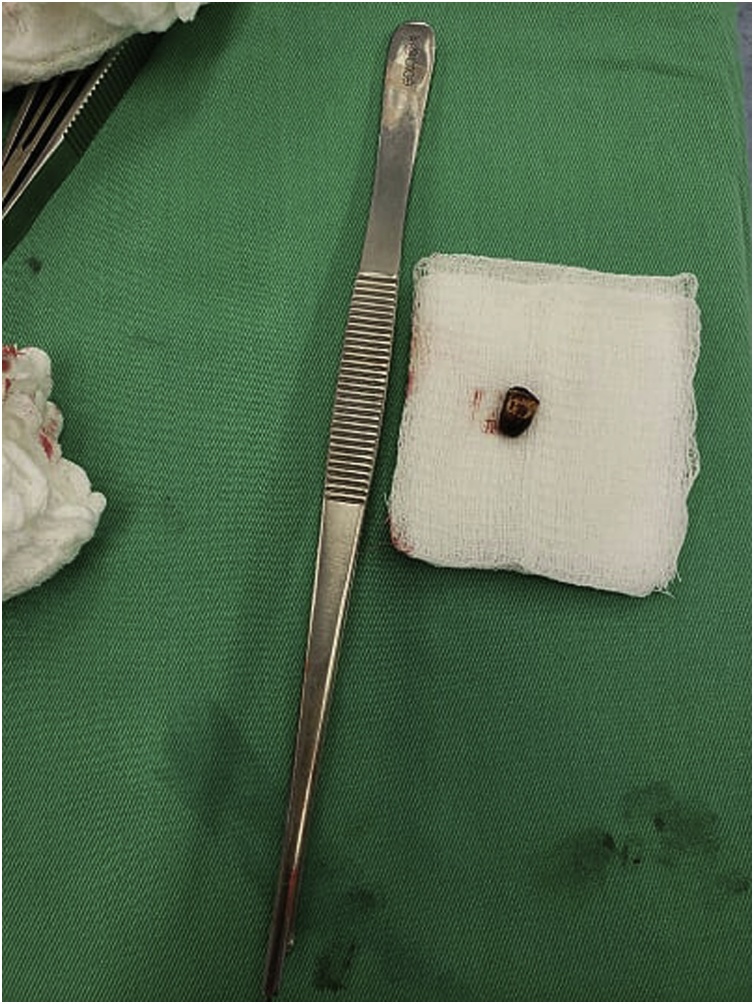
Bullet after the removal.

After being questioned, he refers that one year ago felt like the bullet was moving inside his body. In November of 2018, 45 days after ERCP, it was performed elective surgery for the bullet removal after its visualization by cholangiography in the operating room, in choledochal, through a choledoctomy (Fig. 3, Fig. 4). It was also performed cholecystectomy at the same surgical time. The procedure was a conventional open surgery due to the patient's anatomical difficulties, as it showed scars of previous xifo-pubic median laparotomy, making the use of videolaparoscopy difficult.

The patient was discharged seven days after the surgery, without significant complications and asymptomatic. He has not evolved to new signs or symptoms of cholestatic syndrome or other alterations in the bile ducts until the present moment.

## 3 Discussion

Cholestatic syndrome caused by a gun bullet in a prior event to the symptoms is a very rare event in the literature, with an increase in the number of occurrences during the Second World War and the advent of more accurate image exams and of better visualization [6]. Total, there are 8 case reports of bullets found in bile ducts with distinct evolution time among themselves, varying between 2 months and 9 years, but with similar clinical features [[7], [8], [9], [10], [11], [12], [13], [14]]. Here, we present something new in relation to the other reports; this is the case of jaundice per bullet in bile ducts of longer time until the initial cholestatic symptoms (Table 1), excluding bomb fragments or similar ones [15]. In all the cases, there was prior story of exposition to guns with upper abdomen perforation or in right thoracoabdominal transition. Another similarity among them is the intervention in hepatic parenchyma lesioned by perforation, in which it was not possible to remove the bullet by laparotomy.

**Table 1:** Time until the first cholestatic symptoms and local of bullet impactation.

Article	Bullet impaction Site	Time until first cholestatic symtomps
Krontiris, 1962	Common Bile Duct	7 years
Rescorla, 1996	Common Hepatic Duct	22 months
Simmons, 1998	Common Hepatic Duct	40 months
Maheshwari, 2003	Common Hepatic Duct	2 months
Kamona, 2005	Common Bile Duct	4 months
Hussain, 2007	Common Hepatic Duct	7 years
Stansfield, 2007	Common Hepatic Duct	16 months
Tamasauskas, 2013	Common Hepatic Duct	9 years

Inside the history of traumas per guns and obstructive jaundice, one of the first cases reported in the literature is due to bomb explosion during the Second World War. This case demonstrates the clinical importance during the patient evaluation and the symptoms differences, once the patient only presented fecal acholia [6]. However, this one represents more a historical register once the proceeding undertaken in this case is not performed anymore due to the advancement in diagnosis and practices.

While analyzing the patient clinical status, we see that he occasionally related a complaint similar to biliary colic, what he associated to “bullet movement”, which after all is correct. The object migration, either by the parenchyma or by the bile ducts is well reported in patients with any kind of biliary obstruction by foreign body [7]. We should consider that there is the possibility of complex lesion of the hepatic tree as well as lesion of nearby hepatic vessels. Hypotension and dehydration signs lead us to think in vascular involvement, by either fistula, inflammation or perforation, despite being very rare inside the current literature [13]. Cholangitis can be present although the object nature can alter the microorganism nature, and these ones can colonize the region; besides, just the presence of these elements already increases the chances of infection. We should also consider that the time for the symptoms showing off is not precise, and it could take years for the beginning of the cholestatic picture, as well as it does not define severity, even because most of the patients take longer to become symptomatic [16]. Pancreatitis cases will also give more credit to the hypothesis of foreign body in bile ducts [3,17].

Another important aspect to be detailed is the diagnostic method. Despite classic cholestasis symptoms associated to the history of perforating lesion in typical bile duct lesion region, such as right hypochondrium, by themselves could already define the diagnostics, we need image exams mainly to, besides confirming the hypothesis, visualize the impact location. Initially we can use simple ultrasonography and radiography of the abdomen. The first exam will not necessarily identify the bullet, due to its technical limitations, but it simplifies the visualization of the bile ducts dilation, characterizing obstruction. The simple radiography of the abdomen cannot identify the exact obstruction place, but it will demonstrate if there is metallic object in the place [8]. In case of bullets, it is extremely necessary, because it helps in the diagnosis and it is of easy performance. In case of fragments, it will depend on their size. There are reports of patients with multiple episodes of jaundice, due to fragments not identified in several exams and procedures. We do not recommend the use of magnetic cholangioresonance at the exam, once there is no need of this and it would not present any advantage on the patient behavior, besides it could interfere in the bullet position in the bile duct, causing iatrogenic lesion. In a previous reported case, there was the performance of this exam to confirm the diagnostic, only causing a larger lesion without any change in the case conduct, added to the fact that it was not requested abdomen radiography as first exam [14]. We should always use the simplest exams at the beginning, in case of dubious diagnostic. In case the ultrasonography and the radiography contribute with the patient’s history for jaundice per impact of bullet, the use of endoscopic retrograde cholangiopancreatography (ERCP) should be performed to locate the artifact position more accurately, and if possible, remove the calculus via endoscopy. One observed aspect was that most of the cases presented impact in common hepatic duct, but due to the differences of perforation path, of anatomic bile ducts and of size and format of the bullets, this prevalence in that place does not tend to be relevant. In older cases and with cholangitis installation, it was used the exploration of bile ducts for treatment with the installation of Kehr drain [18]. Nevertheless, whenever possible, primarily use ERCP, once it presents lower complication rates compared to cholangiography and surgical exploration. In case the endoscopist does not feel safe about removing the fragment, they can opt for open track for exeresis [19]. This care with the handling is of extreme importance because the patient, who had already experienced lesion by gun, has already steered away from their daily and labor activities, and a good treatment on this complication generates a faster and satisfactory return to these activities [20].

## 4 Conclusions

The presence of foreign body in the bile ducts is well reported in literature, and it is directly dependent on a specific point that needs to be investigated: previous history. Independently on the incidence of a specific type of findings in bile ducts, the patient’s report will tell us the necessary diagnostic, even more in cases of perforation by gun followed by cholestasis. We should always avoid using cholangioresonance in dubious cases with history compatible with guns, and base ourselves in simpler exams to clarify the diagnosis. To keep the ERCP as initial treatment is recommended due to the advances on the procedure quality, but in more severe cases or in lack of resources, the surgical exploration is still indicated.

## Declaration of Competing Interest

We do not have any conflicts of interests

## Sources of funding

We do not have any funding source, this manuscript is just a case report, not a research

## Ethical approval

As the manuscript is not a research study, we only have the patient consent for writing and others forms of publication. Also, the ethical approval for this case reports has been exempted by our institution

## Consent

Written informed consent was obtained from the patient for publication of this case report and accompanying images. A copy of the written consent is available for review by the Editor-in-Chief of this journal on request.

## Author contribution

Irma C, Ketlen Sousa and Juan Rodriguez made contributions to conception and design. collected the patient details and wrote the paper. Otávio Mendes Filho, Carolina Dorgam and Frank Macedo made contributions to patient management. João Bergamasco, Rubem Silva Neto and Rubem Silva Junior critically revised the article. All authors read and approved the final manuscript.

## Registration of research studies

The manuscript is a case report, not considered a formal research involving participants

## Guarantor

Dr. Frank Macedo

## Provenance and peer review

Not commissioned, externally peer-reviewed

## References

[1] Familiari P., Bulajic M., Mutignani M., Lee L.S. (2005). Endoscopic removal of malfunctioning biliary self-expandable metallic stents. Gastrointest. Endosc..

[2] Khairat M., Allam H.I., Sholiek N., Kassem H. (2005). An unusual common bile duct foreign body. Qatar Med. J..

[3] Kim K., Woo E., Rosato E. (2004). Pancreatic foreign body: ingested toothpick as a cause of pancreatitis and hemorrhage. Gastrointest. Endosc..

[4] Wu W.C., Katon R.M., McAfee J.H. (1991). Endoscopic management of common bile duct stones resulting from metallic surgical clips (eat’s eye calculi). Gastrointest. Endosc..

[5] Agha R.A., Borrelli M.R., Farwana R., Koshy K., Fowler A., Orgill D.P., For the SCARE Group (2018). The SCARE 2018 statement: updating consensus surgical CAse REport (SCARE) guidelines. Int. J. Surg..

[6] Hurt R.L. (1947). Penetrating chest wound with lodgement of the foreign body in the common bileduct. Br. J. Surg..

[7] Hussain S.M.A., Zulqurnain S., Saleem O. (2007). Delayed obstructive jaundice secondary to bullet in common hepatic duct. J. Coll. Physicians Surg..

[8] Kamona A., Mansour A., Qandeel M. (2005). Biliary obstruction secondary to combat-related foreign bodies: report of two cases. Abdom. Imaging.

[9] Krontiris A., Tsironis A. (1962). Common duct obstruction by a bullet compression cap: a rare case. Ann Surg Surg..

[10] Maheshwari M., Chawla A., Dalvi A., Thapar P., Raut A. (2003). Bullet in the common hepatic duct: a cause of obstructive jaundice. Clin. Radiol..

[11] Rescorla F.J., Schlatter M., Hawes R.H., Grosfeld J.L. (1996). Delayed presentation of a penetrating biliary tract injury in a child. J. Trauma.

[12] Simmons T.C., Essilfie W., Fleming A. (1998). Obstructive jaundice occurring 40 months after gunshot wound to the left thoraco-abdomen. Gastrointest. Endosc..

[13] Stansfield W.E., Andreoni K.A. (2007). Peri-operative considerations during biliary exploration and reconstruction: report of delayed biliary obstruction by a bullet. J. Trauma.

[14] Tamasauskas I., Roberto J., Carlotto M., Apodaca F.R., Goldenberg A., Lobo E.J. (2016). Biliary tract obstruction secondary to a foreign body: chambered projectile in common hepatic duct. Relatos Caso do CBC..

[15] Somi M.H., Rezaeifar P. (2010). Shrapnel splinter in the common bile duct. Arch. Iran. Med..

[16] Ban J.L., Hirose F.M., Benfield J.R. (1970). Foreign bodies of the biliary tract: report of two patients and a review of the literature. Ann. Surg..

[17] Danzi J.T. (1986). Two cases of acute pancreatitis due to a foreign body. Gastrointest Endosc [Internet].

[18] Ertuğrul I., Kiliç M., Parlak E., Sahin B. (2006). Foreign body in the common bile duct for 15 years. Gastrointest. Endosc..

[19] Posner M.C., Moore E.E. (1985). Extrahepatic biliary tract injury: operative management plan. J. Trauma.

[20] Roy S.K., Lambert A. (2017). Obstructive jaundice: a clinical review for the UK armed forces. J. R. Nav. Med. Serv..

